# NHERF2 is crucial in ERM phosphorylation in pulmonary endothelial cells

**DOI:** 10.1186/1478-811X-11-99

**Published:** 2013-12-23

**Authors:** Anita Boratkó, Csilla Csortos

**Affiliations:** 1 Department of Medical Chemistry, University of Debrecen Medical and Health Science Center, Egyetem tér 1, Debrecen H-4032, Hungary

**Keywords:** Endothelial cells, Angiogenesis, ERM proteins, NHERF2

## Abstract

**Background:**

EBP50 and NHERF2 adaptor proteins are incriminated in various signaling pathways of the cell. They can bind ERM proteins and mediate ERM-membrane protein interactions.

**Results:**

Binding of ERM to EBP50 and NHERF2 was compared in pulmonary artery endothelial cells by immunoprecipitation. NHERF2 associates with all three ERM, but EBP50 appeared to be a weak binding partner if at all. Furthermore, we detected co-localization of NHERF2 and phospho-ERM at the cell membrane and in the filopodia of dividing cells. Silencing of NHERF2 prevented agonist or angiogenesis induced phosphorylation of ERM, while overexpression of the adaptor elevated the phosphorylation level of ERM, likely catalyzed by Rho kinase 2, which co-immunoprecipitated with NHERF2/ERM in control EC, but did not bind to ERM in NHERF2 depleted cells. Dependence of ERM phosphorylation on NHERF2 was also shown in Matrigel tube formation assay, and NHERF2 was proved to be important in angiogenesis as well. Furthermore, when NHERF2 was depleted or cells were overexpressing a mutant form of NHERF2 unable to bind ERM, we found attenuated cell attachment with ECIS measurements, while it was supported by overexpression of wild type NHERF2.

**Conclusions:**

Pivotal role of NHERF2 in the phosphorylation process of ERM in pulmonary artery endothelial cells is shown. We propose that NHERF2 provides a common anchoring surface for ERM and Rho kinase 2. Our results demonstrate the essential role of NHERF2 in endothelial cell adhesion/migration and angiogenesis.

## Background

The Na^+^/H^+^ exchanger regulatory factor (NHERF) family consists of four scaffolding proteins, namely, NHERF1/EBP50, NHERF2/E3KARP, NHERF3/PDZK1, and NHERF4/IKEPP. They contain two or four PDZ domains which serve as protein-protein interacting sequences [1]. So far, NHERF proteins were mainly investigated in polarized epithelial cells. These studies revealed different locations and different protein binding partners of the members of the family [2,3]. Besides playing an essential role in the regulation of Na^+^/H^+^ exchanger-3 (NHE3), there is a growing body of evidence that NHERF proteins are implicated in many signaling pathways of the cell [4,5].

NHERF1/EBP50 and NHERF2 are similar proteins with 57% amino acid identity and they have the same domain structure [6,7]. Although they have two PDZ domains, they differ from the other members of the NHERF family by having a C-terminal ezrin/radixin/moesin (ERM) binding domain/tail. An important difference between these proteins is that EBP50 is a phosphoprotein. For example, it can be phosphorylated during mitosis by cyclin dependent kinase 1 at two serine residues (Ser279 and Ser301) [8-11], however, NHERF2 does not contain these phosphorylation sites, and phosphorylation of NHERF2 has not been reported yet. Both EBP50 and NHERF2 were described as essential components in the NHE3/ezrin/cAMP dependent protein kinase II multiprotein signaling complex which is required for the inhibition of ion transport via the phosphorylation of NHE3 [12,13]. NHERF2 binds to an internal domain in the cytoplasmic tail of NHE3, and it was suggested that by binding to ezrin, it brings the cAMP dependent protein kinase close enough to the cytoplasmic tail of NHE3, which can be phosphorylated at elevated cAMP levels [7]. A more recent study performed on NHERF2 knockout mice shows that NHERF2 is necessary for normal basal trafficking of NHE3, furthermore, cAMP inhibition and lysophosphatidic acid (LPA) stimulation are NHERF2 dependent. It is also suggested that the effects of NHERF1 and/or NHERF2 in NHE3 regulation are organ and tissue specific [14]. Further binding targets of EBP50 and NHER2 are receptors, scaffolding- and various signaling proteins as reviewed by Shenolikar et al. [15].

ERM proteins are regulated linkers between the plasma membrane and the actin cytoskeleton. They can bind directly to adhesion molecules, but their interaction with membrane proteins can be mediated by adaptor proteins, such as the above mentioned EBP50 and NHERF2 [16]. ERM proteins have similar domain structures, they share an N-terminal FERM domain and the F-actin binding site is in their C-terminal ERM-associated domain (C-ERMAD) [17]. Activation of the ERM proteins is phosphorylation dependent. The head-to-tail intramolecular interaction of inactive ERMs is disrupted by the phosphorylation of a conserved C-terminal threonine residue and the N- and C-terminal domains become available for intermolecular interactions [18,19]. Cell type specific expression of EBP50, NHERF2 and ERM seems to be parallel with the binding preference between the NHERF and ERM proteins [20].

NHERF proteins are less characterized in endothelial cells (EC). Recently, we have shown nuclear localization of EBP50 in the interphase in bovine pulmonary artery endothelial cells (BPAEC) and in HUVEC [21]. During mitosis, phosphorylation and cytoplasmic localization of EBP50 was detected. Furthermore, protein-protein interaction and co-localization with protein phosphatase 2A (ABαC) in mitotic BPAEC have been shown. ECIS (electric cell-substrate impedance sensing) measurements proved that the phosphorylated form of EBP50 supports EC wound healing, suggesting the significance of EBP50 in cell division [21]. Others described NHERF2 as a participant in endothelial homeostasis and vascular remodeling [5].

In the present work binding ability of EBP50 and NHERF2 to ERM was compared in pulmonary artery EC. We show evidence that NHERF2 aids filopodia formation and migration of EC by mediating phosphorylation of ERM by Rho kinase 2 (ROCK2). Our results also indicate that NHERF2 is required for proper EC tube formation.

## Results

### Endothelial EBP50 and NHERF2 have different ERM binding capability

**Figure 1:**
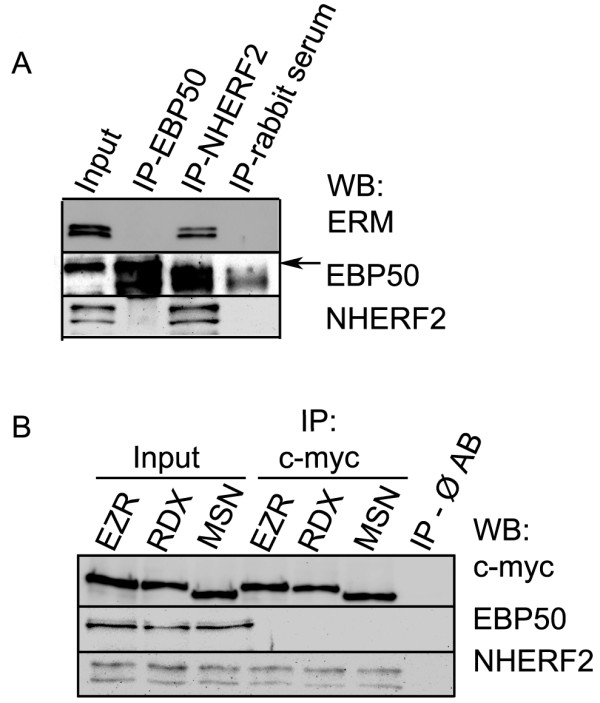
**EBP50 and NHERF2 have distinct interacting partners in EC. (A)** EBP50 or NHERF2 was immunoprecipitated from lysates of BPAEC cells. Total cell lysate (input) and IP complexes were probed for ERM, EBP50 and NHERF2 specific antibodies. **(B)** Anti-c-myc antibody was utilized for immunoprecipitations form pCMV-myc ezrin (EZR), pCMV-myc radixin (RDX) or pCMV-myc moesin (MSN) transfected BPAEC cell lysates as described in Methods. Total cell lysates (input) and IP complexes were probed for c-myc tag, EBP50 and NHERF2. Ø AB: control of IP from BPAEC without the addition of antibody. Shown are representative data of at least 3 independent experiments.

Earlier, we detected both EBP50 and NHERF2 proteins in endothelial cells, however, our results indicated their different subcellular localization, nuclear and cytoplasmic, respectively, in interphase EC [21]. That suggests diverse functions and protein partners of the two adaptors in EC. EBP50 and NHERF2 proteins are known to interact with ERM, as they have ERM binding tails at their C-termini. To study whether endothelial ERM have any distinction between these two adaptor proteins, immunoprecipitation experiments were performed. Endogenous EBP50 and NHERF2 were immunoprecipitated from bovine pulmonary artery EC (BPAEC) lysates and the IP complexes were probed in Western blot with an anti-ERM antibody. As shown in Figure 1A, ERM proteins preferred to bind NHERF2. To decide whether all three ERM are able to bind to NHERF2, mammalian expression constructs were created. BPAEC monolayers were transfected with expression constructs of ezrin, radixin, or moesin, each cloned into pCMV-myc vector. Lysates of the overexpressing cells were subjected to immunoprecipitation with anti-c-myc antibody. Total cell lysates, to confirm the overexpression of ezrin, radixin, or moesin, and the IP complexes were tested in Western blot with monoclonal anti-c-myc, EBP50 and NHERF2 antibodies. While EBP50 was not detectable in these IP samples, the endogenous NHERF2 co-immunoprecipitated with each of the recombinant ERM proteins (Figure 1B). Therefore we have focused our further investigation on the ERM-NHERF2 interaction.

### Phospho-ERM binds to NHERF2

**Figure 2:**
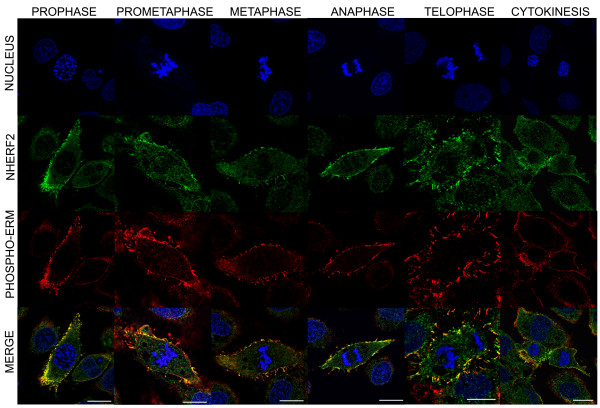
**Co-localization of phospho-ERM and NHERF2 during mitosis in BPAEC.** Immunofluorescence staining of BPAEC was performed using anti-phospho-ERM (red) and anti-NHERF2 (green) primary antibodies. Phases of the cell cycle were identified using TO-PRO-3 Iodide staining. Scale bars: 10 μm. Shown are representative data of at least 3 independent experiments.

Protein-protein interaction of NHERF2 and the phosphorylated form of ERM proteins was analyzed by immunofluorescent staining. BPAEC cells were co-stained with anti-phospho-ERM and anti-NHERF2 antibodies. Nuclei were visualized by TO-PRO-3 Iodide. We observed co-localization of the two proteins in the cell membrane and filopodia of dividing cells at all phases of mitosis (Figure 2). These results imply that NHERF2 may bind to phospho-ERM proteins during mitosis.

**Figure 3:**
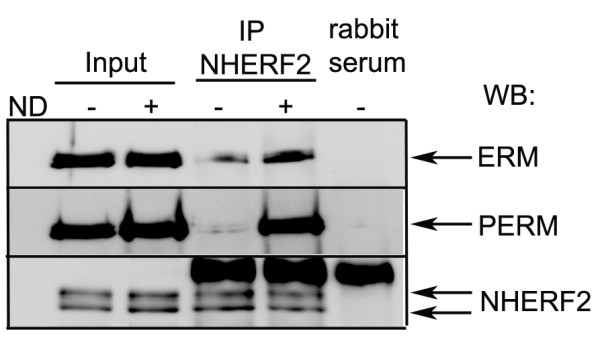
**Phospho-ERM co-immunoprecipitates with NHERF2 from mitotic EC.** NHERF2 was immunoprecipitated from lysates of BPAEC without or with nocodazole (ND) treatment as described in Methods. Cell lysates (input) and IP complexes were probed for ERM, phospho-ERM and NHERF2. Shown are representative data of at least 3 independent experiments.

Next, cells were arrested in G2/M phase by 80 ng/ml nocodazole treatment for 16 h to induce phosphorylation of ERM in large number of the cells, and then NHERF2 was immunoprecipitated. Lysates of control and nocodazole treated cells as well as the IP complexes were probed with antibodies against ERM, phospo-ERM and NHERF2 in Western blot (Figure 3). Indeed, the nocodazole challenge increased the phosphorylation level of ERM compared to the asynchronized cells. In addition, greater amount of phospho-ERM was detected in NHERF2 immunoprecipitates after nocodazole.

### NHERF2 mediates phosphorylation of ERM through interaction with ROCK2

**Figure 4:**
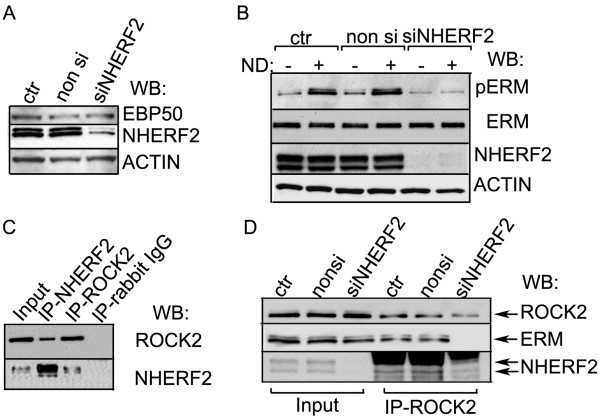
**ERM cannot be phosphorylated in the absence of NHERF2. (A)** Lysates of non transfected (ctr), non-siRNA or NHERF2 specific siRNA (sc-42522) treated cells were analyzed by Western blot using anti-EBP50, -NHERF2 and -actin antibodies. **(B)** Lysates of non transfected (ctr), non-siRNA or NHERF2 specific siRNA (sc-42522) treated cells without or with nocodazole (ND) challenge were analyzed by Western blot using antibodies against phospho-ERM, ERM, NHERF2 and actin as described in Methods. **(C)** NHERF2 or ROCK2 was immunoprecipitated from lysates of BPAEC. The IP complexes were probed for ROCK2 and NHERF2. **(D)** ROCK2 was immunoprecipitated from lysates of control, non-siRNA or NHERF2 specific siRNA treated cells. IP complexes were probed for ROCK2, ERM and NHERF2. Shown are representative data of at least 3 independent experiments.

To check the possible regulatory role of NHERF2 adaptor protein in ERM phosphorylation, NHERF2 (SLC9A3R2) was depleted in BPAEC cells using specific silencing RNA duplexes. Five different siRNAs against NHERF2 were tested, and the two most efficient ones (sc-42522 and SI03084977) were used. The efficiency of depletion was checked by Western blot (Figure 4A). Silencing of NHERF2 did not change the protein level of EBP50 (Figure 4A). Lysates of control, non silencing RNA and NHERF2-specific silencing RNA transfected cells without or with nocodazole treatment were analysed by Western blot using antibodies against phospho-ERM, ERM, NHERF2, and actin (Figure 4B and Additional file 1: Figure S1). The phosphorylation level of ERM was about the same in control and non silencing RNA transfected cells without additional effector, and it increased to the same extent after the nocodazole treatment. More importantly, the phosphorylation level of ERM was extremely low in NHERF2 depleted cells and did not increase in dividing cells or after the nocodazole challenge (Figure 4B and Additional file 2: Figure S2). These results imply that the adaptor protein is a necessary factor for ERM phosphorylation.

One reasonable explanation of this observation can be that NHERF2 provides a common binding surface for both the ERM and the protein kinase which phosphorylates ERM. The increase in phosphorylation level of ERM evoked by nocodazole was significantly attenuated in the cells which were pretreated with H1152 to inhibit ROCK2 (not shown). To further test this hypothesis, Rho kinase 2 (ROCK2) and NHERF2 were immunoprecipitated from BPAEC lysates and in fact, both ROCK2 and NHERF2 were present in the two immunoprecipitates (Figure 4C). Furthermore, we could detect ERM and NHERF2 in ROCK2 IP complexes from control and non-siRNA transfected EC, but the ERM was not present in the ROCK2 immunoprecipitate from NHERF2 depleted cells (Figure 4D) suggesting the plausibility of our assumption.

### NHERF2 aids EC filopodia formation and cell spreading

**Figure 5:**
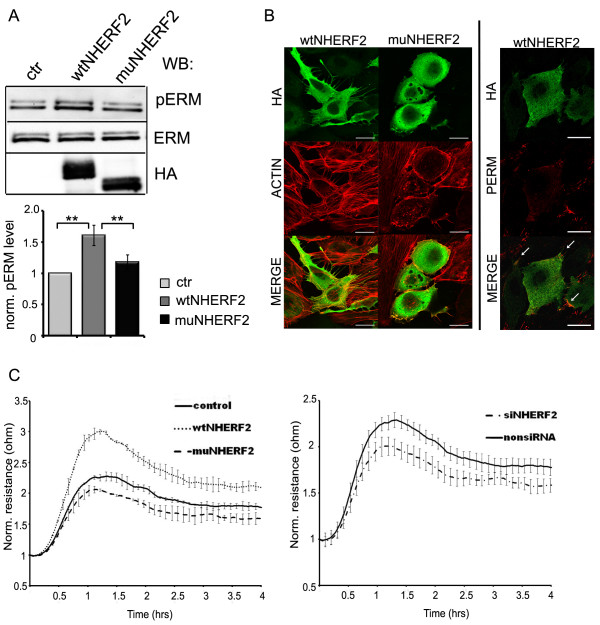
**NHERF2 overexpression induces EC filopodia formation and spreading. (A)** Lysates of BPAEC without transfection (ctr) or transfected with pCMV-HA wild type or mutant NHERF2 constructs were analyzed by Western blot using antibodies against phospho-ERM, ERM, and HA-tag. A representative Western blot is shown. Protein levels were quantified by densitometric analysis. Phospho-ERM protein levels were normalized against ERM protein levels. Bars represent mean ± SE. Significant changes, determined by Student’s *t-*test, are indicated by asterisks; **(P < 0.01), n = 6. **(B)** Left panel: Immunofluorescent staining of recombinant wild type or mutant NHERF2 overexpressing BPAEC was performed using anti-HA primary antibody (green). Actin microfilaments were stained with Texas Red conjugated phalloidin (red). Scale bars: 10 μm. Right panel: Wild type NHERF2 transfected BPAEC co-stained for phospho-ERM and HA-tag is shown. Arrows indicate co-localization of phosho-ERM and overexpressed NHERF2 on the merged image. **(C)**: Non transfected (control), wild type (wt) and mutant (mu) NHERF2 transfected cells (left panel) or non-siRNA and NHERF2 specific siRNA treated cells (right panel) were plated onto 8W10E arrays. Spreading and attachment of cells were followed in time by ECIS measurement. Results are presented as means ± SD at least of four chambers for each sample.

In accordance with the above observations, NHERF2 overexpression increased the phosphorylation level of ERM. The entire coding region of NHERF2 was amplified by RT-PCR and it was cloned into a pCMV-HA vector. Another construct, producing a truncated mutant form of NHERF2, was also created. The mutant misses the C-terminal ERM-binding tail, therefore it is not able to bind to ERM. BPAEC were transfected with these constructs, and the effects of the overexpressed proteins on phospho-ERM level were analyzed by Western blot. Overexpression of wild type (wt) NHERF2 resulted in an increased phosphorylation level (about 50% increase) of ERM, while the mutant NHERF2 lacking the ERM-binding domain did not trigger a significant increase in that (Figure 5A). Immunofluorescent staining revealed that cells overexpressing wtNHERF2 show strong filopodia formation compared to non-overexpressing EC, or cells transfected with the mutant NHERF2 and the recombinant NHERF2 co-localizes with phospho-ERM (Figure 5B).

As filopodia play a role in cell migration and adhesion, to monitor cell spreading and attachment ECIS measurements were utilized. Sufficient number of EC transfected with wt- or mutant NHERF2 were plated onto 8W10E arrays 24 h post-transfection to form confluent monolayers and the resistances of the ECIS electrodes were followed in time. The more rapid spreading dynamics of wtNHERF2 overexpressing cells compared to the control or mutant NHERF2 overexpressing cells is clearly apparent (Figure 5C, left panel). In a parallel ECIS experiment, non-siRNA and NHERF2-specific siRNA transfected EC were compared. As expected, the barrier formation of NHERF2 depleted cells was slower than that of for the non siRNA treated cells (Figure 5C, right panel).

### NHERF2 affects EC tube formation

**Figure 6:**
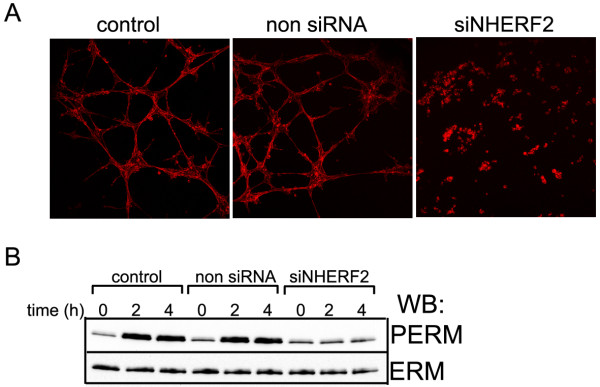
**NHERF2 is required for EC tube formation. (A)** Control, non-siRNA or silencing RNA specific for NHERF2 transfected BPAEC were seeded on Matrigel-coated μ-Slide plates. F-actin staining was done at 5 h after seeding, and images were captured by confocal microscopy. Silencing of NHERF2 inhibits cord formation, resulting in the formation of cell aggregates in the Matrigel. **(B)** Western blot analysis of ERM and phospho-ERM in control, non-siRNA or NHERF2 specific silencing RNA transfected BPAEC grown in Matrigel and processed at 0, 2 and 4 h after seeding.

Endothelial cell migration and proliferation are essential in angiogenesis. Therefore, based on the above results, we hypothesized that through the control of the phosphorylation level of ERM proteins, NHERF2 plays a regulatory role in angiogenesis. Control, non-siRNA- and NHERF2-specific siRNA transfected EC were seeded on μ-Slide plates coated with Matrigel. These tube formation assays were monitored for 8 h with a light microscope. Control cells started to form polygon structures at about 3 h after seeding, and the network formation reached a peak at 5 h. Silencing of NHERF2 inhibited the network formation and resulted in formation of cell aggregates in the Matrigel (Figure 6A). Next, ERM and phospho-ERM levels in lysates of control, non-siRNA- and NHERF2-specific silencing RNA transfected cells - seeded onto Matrigel - were analyzed before and during the course of the tube formation assays (Figure 6B). The phosphorylation level of ERM proteins increased greatly in control and non-siRNA treated cells without any change in the ERM protein level. In contrast, the phosphorylation level of ERMs in NHERF2 silenced cells could not change. Our results indicate that NHERF2 is a crucial component in EC tube formation by supporting the phosphorylation process of ERM.

## Discussion

Regulation of endothelial cytoskeleton structure remodeling is essential in angiogenesis and in development and maintenance of vascular barrier; and eventually, in proper lung function. ERM are actin-binding linkers connecting F-actin and the plasma membrane, either directly or indirectly via adaptor proteins. NHERF scaffolding proteins are known to be regulators of NHE3 in epithelial cells, but they are also common interacting partners of ERM proteins [1,15,22,23]. Based on the primary sequence and the domain structure, NHERF1/EBP50 and NHERF2/E3KARP are the most similar members of the family [6,7,22], both proteins have two PDZ domains and an ERM binding domain at their C-termini. Previously, we have shown cell-cycle and phosphorylation dependent localization of EBP50 in EC in the nucleus [21], NHERF2, on the other hand, did not appear in the nuclei of the immunostained EC. The present work indicates that ERM binds preferentially NHERF2 over EBP50 in EC. It was reported that in HUVEC cells NHERF2 but not EBP50 is highly expressed [5]. However, we cannot explain the difference with lower protein level of EBP50, as semiquantitative assessment of our RT-PCR products produced with EBP50 and NHERF2 specific oligonucleotide primers indicated similar expression levels of the two adaptors in pulmonary artery EC (data not shown). These results suggest that NHERF2 has not only different binding partners, but also its function(s) can be unlike that of EBP50 in EC. Studies of NHERF localizations and functions in other cell types also demonstrated such diversity. EBP50, for instance, is the most enriched in tissues with extensive, polarized epithelia and it is localized in cell surface microvilli. ERM and EBP50 were reported to co-localize in the cell surface, preference for ERM-EBP50 interaction depending on tissue and cell type was also proposed [22]. Cell type specific appearance was observed in kidney cells, EBP50 being abundant in proximal tubule cells, while NHERF2 was detected in the glomerulus [2]. Another work also claims tissue-specific expression of the two adaptor proteins and suggests EBP50-ezrin vs NHERF2-moesin/radixin pairing [20]. Our studies with the pulmonary artery EC did not indicate distinct abundancy of the two adaptors or pairing among EBP50 or NHERF2 and the individual ERM proteins. However, our findings imply that NHERF2 is the preferred partner of ERM over EBP50.

Therefore, major part of this work was focused on the investigation of the NHERF2-ERM complex in pulmonary aorta EC. We found that phospho-ERM and NHERF2 co-localize at the cell membrane and in the filopodia in dividing EC, moreover, phospho-ERM was present in NHERF2 IP. ERM and NHERF2 are known to bind and bring together membrane and non-membrane proteins, providing structural links and organizing proteins, and that may result in their involvement in several signal transduction pathways. Role of ERM in RhoA, PKA, insulin, or membrane receptor signaling, development, differentiation, migration etc. have been reported [24-26]. Various binding partners of NHERF2 including virulence factors, Map, EspI and NleH1 [27], EPI64, a microvillar protein [28], LPA2 receptor [29], or β-catenin [30] advocate broad functions of the adaptor beside the regulation of NHE3. Our results suggest that the ERM/NHERF2 protein-protein interaction may have an importance in the phosphorylation process of ERM, and consequently, NHERF2 can be significant in cytoskeleton remodeling of EC. Both depletion and overexpression of NHERF2 proved the above assumption. When NHERF2 was silenced, nocodazole treatment could not evoke ERM phosphorylation; on the other hand, overexpression of NHERF2 increased the phospho-ERM level.

Ezrin, radixin and moesin are activated by phosphorylation of a threonine residue (T576, T564, T558, respectively) [25]. Several kinases can phosphorylate ERM on this threonine, including ROCK2 [18,25]. Our results imply that NHERF2 is a key player in ERM phosphorylation by presenting binding surface for ERM and ROCK2. Although it is not completely clear yet whether both ERM and ROCK2 bind directly to NHERF2, one may assume attachment of ERM to the C-terminal ERM binding domain of NHERF2. Direct contact between NHERF2 and the kinase cannot be excluded based on our results. ROCK2 contains a pleckstrin-homology like (PH) domain [31] which may interact with one of the PDZ domains of NHERF2 [32]. The SRL amino acid sequence at the N-terminal part of the PH domain in ROCK2 fits a recognition motif, S/T-X-I/V/L, reported for NHERF2, although this motif is usually positioned at the C-terminal of the PDZ-binding peptide [29,33]. Although further research is required to elucidate this suggestion, our finding that ERM was not present in ROCK2 IP from NHERF2 depleted cells fits into this concept.

In NHERF2 overexpressing cells we detected increased ERM phosphorylation level and enhanced filopodia formation. A relevant finding of Gandy et al. [34] indicates a similar linkage between ERM phosphorylation and filopodia formation in HeLa cells. Moreover, Theisen et al. [30] showed that HT1080 fibrosarcoma cells expressing a PDZ-domain mutant form of NHERF2 have reduced lamellipodia and impaired cell migration, indicating the substantial regulatory role of NHERF2 in cell migration.

An interesting study of Bhattacharya et al. [5] on HUVEC reports an increase in endothelial proliferation after NHERF2 knockdown and a critical role of NHERF2 in endothelial homeostasis. Nevertheless, it has to be noted that in our experiments overexpression or silencing of NHERF2 in pulmonary artery EC did not cause detectable change in the proliferation rate of cells. This indicates the possibility of an altering role of NHERF2 in different endothelial cell types.

Our results demonstrate that EBP50 and NHERF2 not only have different localizations in vascular endothelial cells, but they have differing functions in these cells as well. In the NHERF2 depleted cells the protein level of EBP50 did not increase which could be the sign of substitution in function, instead, we observed alterations in phospho-ERM level and filopodia formation during mitosis. Therefore we suggest that NHERF2 is an essential binding partner of ERM that aids phosphorylation of ERM and eventually is involved in the arrangement/rearrangement of plasma membrane-ERM-actin bridges during filopodia formation and cell division. EBP50, on the other hand, may have other binding partner(s) in the nuclei of these cells and it may contribute in the transfer of those partner(s) to cytoplasmic locations, cytoskeletal elements during mitosis. Similarly, while there is an overlap in the binding partners of EBP50 and NHERF2 in epithelial cells like NHE3, there are also evidences indicating their unique specificity for protein partners and cellular functions [14,35]. Interestingly, although an increase of NHERF2 protein level in EBP50(−/−) kidney membrane fraction was observed, still, the ERM/P-ERM level was decreased in the membrane suggesting the pivotal role of EBP50 in organizing apical epithelial membranes [36].

The effects of NHERF1 and 2 knockouts were studied on epithelial cells in relation to the intestinal ion transport by Seidler’s group [37-39]. They report different roles of EBP50 and NHERF2 in regulating cystic fibrosis transmembrane conductance regulator (CFTR). Recent studies of Song et al. [40,41] describe effects of EBP50 knockout on migration and proliferation of vascular smooth muscle cells. They suggest that EBP50 is a key regulator of vascular remodeling as they found EBP50 to be required in neointima formation following endoluminal injury in mice.

We investigated the possible involvement of NHERF2 in major physiological functions of EC. ERM proteins participate in cell adhesion and migration [24,42], which are key components of barrier formation and angiogenesis. Our results point to the critical role of phosphorylation of ERM aided by NHERF2 in cell adhesion and migration, as cell spreading and attachment of barrier forming EC was attenuated in NHERF2 depleted cells or mutant NHERF2 (without the ERM binding domain) overexpressing EC. On the other hand, overexpression of wild type NHERF2 resulted in a faster cell spreading compared to the controls. Furthermore, NHERF2 may affect angiogenesis as well, as we have shown that the polygonal network formation of NHERF2 depleted cells in Matrigel was inhibited. Our results imply the role of ROCK2 in these processes. Participation of ROCK activity in endothelial barrier maintenance was reported earlier in connection with the EC junctions [43]. They claim that ROCK has a dual role in regulation of EC barrier function, a protective activity at the cell margins and a barrier-disruptive activity at contractile F-actin stress fibers. It is also important to note that ezrin hyperphosphorylation was found to correlate with invasiveness of HCC (hepatocellular carcinoma), and inhibition of ROCK activity reduced ezrin phosphorylation and resulted in a blockade to HCC cell invasion [44]. Together with our new results it raises the question whether NHERF2, as a modulator of ERM phosphorylation via ROCK2, may affect invasiveness of carcinoma cells.

## Conclusions

In summary, NHERF2 is the preferred ERM-binding partner over EBP50 in pulmonary aorta EC. Our results advocate the existence of an NHERF2-ERM-ROCK2 linkage which seems to be critical in filopodia formation and cell spreading, and consequently in EC barrier formation and angiogenesis.

## Methods

### Reagents

Materials were obtained from the following sources: Ezrin/Radixin/Moesin antibody, Phospho-Ezrin (Thr567)/Radixin (Thr564)/Moesin (Thr558) antibody, HA-tag rabbit mAb, ROCK2 rabbit mAb, anti-rabbit IgG HRP-linked and anti-mouse IgG HRP-linked secondary antibodies: Cell Signaling Technology, Inc. (Beverly, MA); anti-NHERF2 (C-2) antibody: Santa Cruz Biotechnology, Inc. (Santa Cruz, CA); anti-SLC9A3R1 antibody (NHERF1/EBP50): Abgent (San Diego, CA); monoclonal anti-c-myc antibody: Zymed Laboratories (South San Francisco, CA); Alexa 488-, Alexa 594-conjugated secondary antibodies and ProLong Gold Antifade medium with DAPI: Molecular Probes (Eugene, OR), Protease Inhibitor Cocktail Set III: EMD Biosciences (San Diego, CA); pCMV-HA and pCMV-myc vectors: Clontech Laboratories, Inc. (Mountain View, CA). Substances for cell culturing were from PAA (Austria). Anti-SLC9A3R2, anti-actin antibody and all other chemicals were obtained from Sigma (St Louis, MO).

### Cell cultures

Bovine pulmonary artery endothelial cells (BPAEC) (culture line-CCL 209) were obtained frozen at passage 8 (American Type Tissue Culture Collection, Rockville, MD), and were utilized at passages 15–20. Cells were maintained at 37°C in a humidified atmosphere of 5% CO_2_ and 95% air in MEM supplemented with 10% (v/v) heat inactivated fetal bovine serum, 1% sodium pyruvate, 0.1 mM MEM non-essential amino acids solution.

### Preparation of vector constructs

The coding region of NHERF2 (NM_001077065.1), ezrin (NM_003379.4), radixin (NM_002906.3), moesin (NM_002444) and neurofibromin2 (NM_000268.3) was amplified by RT-PCR using the following primers. Ezrin forward: 5′-AAGAATTCCCATGCCGAAACCAATCAA-3′, reverse: 5′-GGCTCGAGTTACAGGGCCTCGAA-3′; radixin forward: 5′-TCGTCGACCATGCCGAAACCAATCAACGT-3′, reverse: 5′-TATGCGGCCGCTCACATTGCTTCAAACTCAT-3′; moesin forward: 5′- AAGAATTCCCATGCCCAAAACGATCAGT-3′, reverse: 5′- GGCTCGAGTTACATAGACTCAAATTCGTC-3′; NHERF2 wild type forward: 5′-GAGAATTCTTATGGCCCGCTCTGGGAAT-3′, reverse: 5′-TCCTCGAGTCAGAAGTTGCTGAAGATCTC-3′; NHERF2 mutant forward: 5′-GAGAATTCTTATGGCCCGCTCTGGGAAT-3′, reverse: 5′-AACTCGAGCTACTGAAAAGGATCTCGCTTCC-3′. All primers were synthesized by Integrated DNA Technologies (Coralville, IA). The PCR products were subcloned into pCMV-myc (ERM) or pCMV-HA (NHERF2) mammalian expression vectors using restriction sites created by the PCR primers. The DNA sequences of the constructs were confirmed by sequencing (Clinical Genomics Center, MHSC, RCMM, University of Debrecen).

### Transfection, siRNA silencing

BPAEC cells were transfected with pCMV-myc ezrin, pCMV-myc radixin, pCMV-myc moesin or pCMV-HA NHERF2 wild type and mutant plasmids using Lipofectamine 2000 transfection reagents (Invitrogen Corporation, Carlsbad, CA), according to the manufacturer’s instructions. After 24 hours cells were washed and lysed.

NHERF2 (SLC9A3R2) was silenced using 25 nM NHERF2 specific siRNA (sc-42522 from Santa Cruz or SI00068376, SI00068383, SI03075562 and SI03084977 from Qiagen) in complex with DharmaFECT-1 transfection reagent (Dharmacon) in serum-free medium. ON-TARGETplus siCONTROL nontargeting pool (D-001810-10-01-05; Dharmacon) was used as an irrelevant control. After 6 h the medium was changed to complete medium. Cells were further incubated for 48–72 hours. The two most efficient siRNA (sc-42522 and SI03084977) were used.

### Immunofluorescence and microscopy

Cells were grown on glass coverslips, washed once with 1X TBS and fixed with 3.7% paraformaldehyde in 1X TBS for 10 min. Between each step, the cells were rinsed three times with 1X TBS. All steps were performed at room temperature. The cells were permeabilized with 0.5% Triton X-100 in TBS for 15 min, blocked with 2% BSA in TBS for 30 min, and incubated with primary, then with secondary antibodies diluted in blocking solution for 1 h. Coverslips were rinsed and mounted in ProLong Gold Antifade medium. Confocal images were acquired with an Olympus Fluoview FV1000 confocal microscope using UPLSAPO 60x 1.35 NA oil immersion objective on an inverted microscope (Olympus IX81) or with a Leica TCS SP8 confocal microscope using HC PL APO CS2 63x 1.40 NA oil immersion objective on an DMI6000 CS microscope at 25°C. Images were processed using FV10-ASW v1.5 or LAS AF v3.1.3 software. Nonspecific binding of the secondary antibodies was checked in control experiments (not shown).

### Immunoprecipitation

Cells (~1×10^7^) grown in 10 cm Ø dishes were rinsed three times with 1x PBS and then collected and lysed with 600 μl of immunoprecipitation (IP) buffer (20 mM Tris HCl, pH 7.4, 150 mM NaCl, 2 mM EDTA, 2 mM sodium vanadate, 1% Nonidet P-40) containing protease inhibitors. The lysates were centrifuged with 10,000 *g* for 15 min at 4°C. To avoid nonspecific binding, the supernatants were precleared with 50 μl of protein G Sepharose (GE Healthcare, Piscataway, NJ) at 4°C for 3 h with end-over-end rotation. Protein G Sepharose was removed by centrifugation at 4°C for 10 min, and the supernatant was incubated with the appropriate volume of antibody (5 μg) at 4°C for 1 h and then with 50 μl of fresh protein G Sepharose at 4°C overnight with gentle rotation. The resin was washed three times with 300 μl of IP buffer and then resuspended in 150 μl of 1X SDS sample buffer, boiled, and microcentrifuged for 5 minutes. The supernatant (20 μl) was further analyzed by Western blot.

### Western blotting

Protein samples (20 μg total protein each) were separated by SDS-PAGE and transferred to 0.45 μm pore sized Hybond ECL Nitrocellulose Membrane (GE Healthcare, Piscataway, NJ). Western blots were imaged using an Alpha Innotech FluorChem® FC2 Imager or Kodak Medical X-ray Developer.

### ECIS measurements

ECIS (Electric cell-substrate impedance sensing) model Zθ, Applied BioPhysics Inc. (Troy, NY) was used to monitor spreading and attachment of control or transfected cells seeded on type 8W10E arrays.

### *In vitro* tube formation assay

BD Matrigel™ Basement Membrane Matrix (BD Biosciences) was used to study the effect of NHERF2 silencing on BPAEC capillary tube formation in accordance with the manufacturer’s instructions. Control, non silencing RNA or NHERF2-specific siRNA treated BPAEC (~1 × 10^3^cells) were plated in μ-Slide (Ibidi, Germany) previously coated with Matrigel and incubated in triplicates at 37°C. Samples were fixed with 2% paraformaldehyde for 10 min, permeabilized with 0.5% Triton-X for 20 min and blocked with 2% BSA in TBS for 20 min. Each step was made at room temperature. CF594 conjugated phalloidin (Biotium, Inc. Hayward, CA) was used to visualize actin filaments. Representative photomicrographs of tube formation from each group were captured by Leica TCS SP8 microscope using HC PL FLUOTAR 10x 0.30 NA objective.

## Abbreviations

BPAEC: Bovine pulmonary artery endothelial cells; C-ERMAD: C-terminal ERM-associated domain; EC: Endothelial cell; ECIS: Electric cell-substrate impedance sensing; ERM: Ezrin/Radixin/Moesin; IP: Immunoprecipitation; NHE3: Na^+^/H^+^ exchanger-3; NHERF: Na^+^/H^+^ exchanger regulatory factor; ROCK2: Rho kinase 2; Wt: Wild type.

## Competing interests

The authors declare that they have no competing interests.

## Authors’ contributions

AB planned and carried out the experiments and made evaluations of results, drafted the manuscript. CC planned the experiments, made evaluations of results and wrote the paper. Both authors read and approved the final manuscript.

## Supplementary Material

Additional file 1: Figure S1 Lysates of non transfected (ctr), non-siRNA or NHERF2 specific siRNA (SI03084977) treated cells without or with nocodazole (ND) challenge were analyzed by Western blot using antibodies against phospho-ERM, ERM, NHERF2 and actin as described in Methods. Click here for file

Additional file 2: Figure S2 Non silencing RNA or NHERF2 specific siRNA transfected BPAEC cells were immunostained with anti-NHERF2 (green) and anti-phospho-ERM (red) antibodies. Nuclei were visualized using TO-PRO-3-Iodide. Click here for file
