# Supplementary figures and images for: NHERF2 is crucial in ERM phosphorylation in pulmonary endothelial cells
Source: Cell Commun Signal. 2013 Dec 23;11:99. doi: 10.1186/1478-811X-11-99 (PMC3880038; doi:10.1186/1478-811X-11-99)

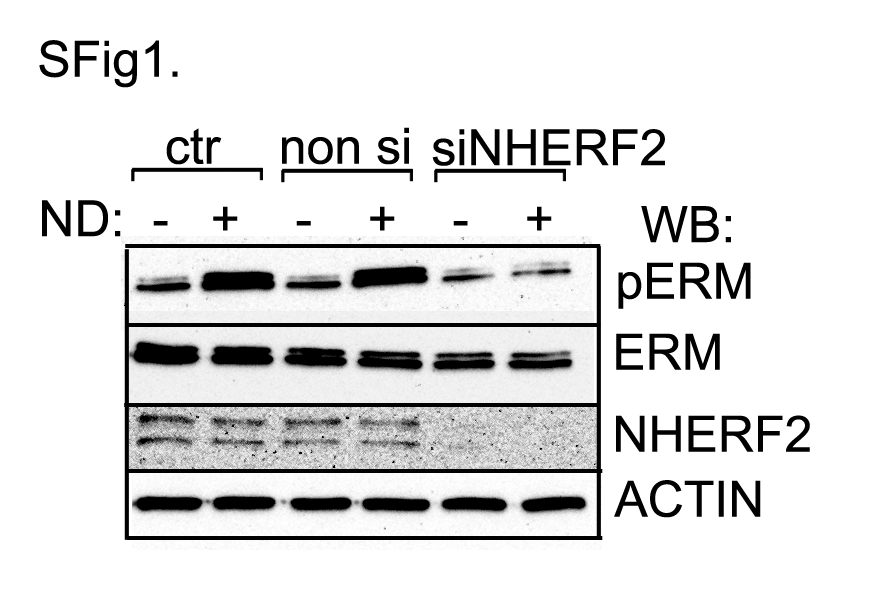

Supplement: Additional file 1: Figure S1 — Lysates of non transfected (ctr), non-siRNA or NHERF2 specific siRNA (SI03084977) treated cells without or with nocodazole (ND) challenge were analyzed by Western blot using antibodies against phospho-ERM, ERM, NHERF2 and actin as described in Methods. [file 1478-811X-11-99-S1.tiff]

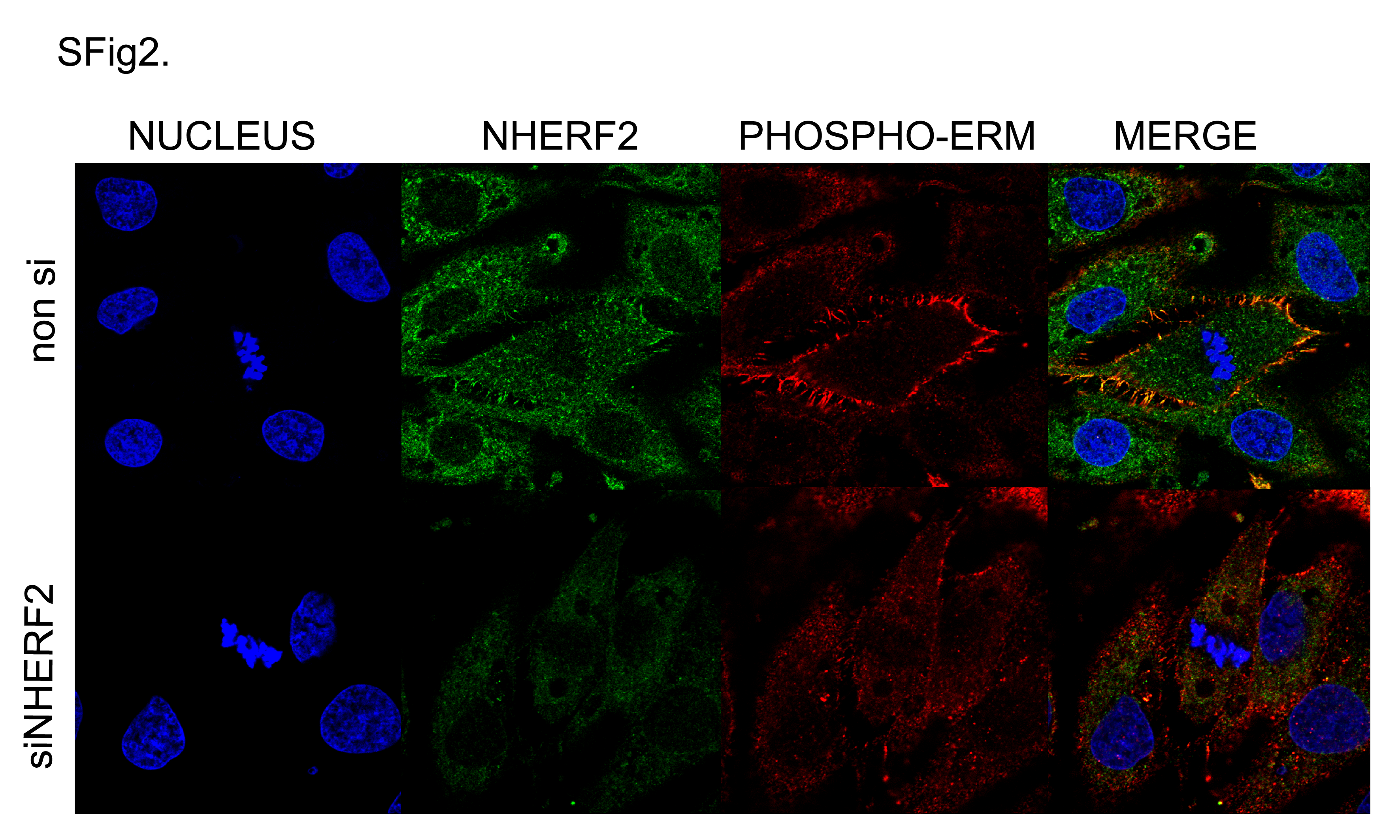

Supplement: Additional file 2: Figure S2 — Non silencing RNA or NHERF2 specific siRNA transfected BPAEC cells were immunostained with anti-NHERF2 (green) and anti-phospho-ERM (red) antibodies. Nuclei were visualized using TO-PRO-3-Iodide. [file 1478-811X-11-99-S2.tiff]
